# Nitazenes: The Emergence of a Potent Synthetic Opioid Threat

**DOI:** 10.3390/molecules30193890

**Published:** 2025-09-26

**Authors:** Joana R. P. Pereira, Alexandre Quintas, Nuno R. Neng

**Affiliations:** 1 Laboratório de Ciências Forenses e Psicológicas Egas Moniz, Quinta da Granja, 2829-511 Caparica, Portugal; jrpereira@egasmoniz.edu.pt (J.R.P.P.); aquintas@egasmoniz.edu.pt (A.Q.); 2 Centro de Química Estrutural, Institute of Molecular Sciences, Departamento de Química e Bioquímica, Faculdade de Ciências, Universidade de Lisboa, 1749-016 Lisbon, Portugal; 3 Egas Moniz Center for Interdisciplinary Research, Egas Moniz School of Health & Science, Quinta da Granja, 2829-511 Caparica, Portugal

**Keywords:** nitazenes, synthetic opioids, forensic toxicology, public health, harm reduction, drug checking

## Abstract

The global unregulated drug supply faces a critical challenge with the emergence of nitazenes, a class of novel synthetic opioids (NSOs) structurally distinct from fentanyl and associated with extreme potency and high risk of fatal overdose. First synthesised in the late 1950s, etonitazene was a target of preclinical research in rats and rhesus monkeys, but it never reached clinical trials due to an unfavourable balance between therapeutic and toxic effects. Nitazenes’ consistent reappearance began in 2019 with isotonitazene, followed by a rapid proliferation of analogues worldwide, many reported to be hundreds to thousands of times more potent than morphine and, in some cases, stronger than fentanyl. This rise is fuelled by their ease of synthesis, low production costs, and evasion of regulatory controls. Nitazenes are frequently mis-sold as counterfeit medications or adulterated into other drugs, resulting in unintentional exposure and overdose, particularly among opioid-naïve users. The primary cause of death is severe and prolonged respiratory depression. Analytical challenges are significant, as traditional screening methods are ineffective, and the low concentration in biological samples requires expensive and highly sensitive liquid chromatography mass spectrometry techniques. This perspective paper highlights critical gaps in detection, clinical management, and regulatory readiness for nitazenes. Urgent efforts are needed to improve surveillance, develop robust analytical methodologies, provide clinical guidance to nitazene intoxications, and strengthen international policy to curb their proliferation.

## 1. Introduction

The global unregulated drug supply is facing an increasingly urgent and dynamic challenge with the emergence of novel synthetic opioids (NSOs), particularly the class known as nitazenes. These compounds are considered a significant public health threat due to their extreme potency, rapid evolution, and the severe risks they pose, including fatal overdoses [1,2,3].

**Figure 1:**
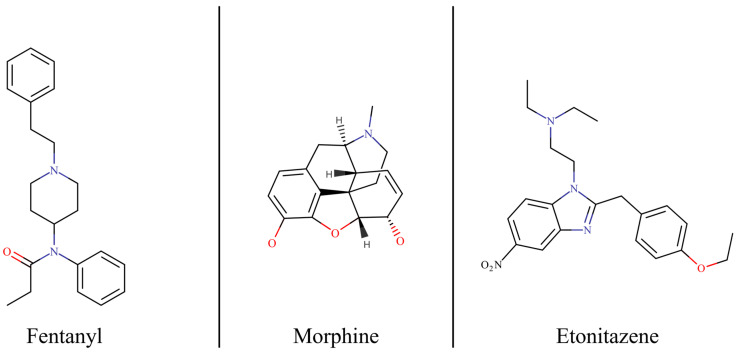
Chemical structure of fentanyl, morphine and etonitazene. Marvin was used for drawing the chemical structures: Marvin 17.21.0, Chemaxon (https://www.chemaxon.com).

Nitazenes are 2-benzylbenzimidazole opioids first synthesised in the late 1950s by CIBA AG pharmaceutical company during the search for new analgesics. Despite exhibiting strong analgesic effects, none of the nitazene opioids were ever approved for clinical use due to significant safety concerns and a high risk of adverse side effects such as severe respiratory depression and high addictive potential. This class of synthetic opioids, shown in Figure 1, is structurally distinct from both traditional morphine-like phenanthrenes and meperidine analogues like fentanyl [4].

Before 2019, nitazenes were primarily known only to researchers studying opioid pharmacology, except for a report of recreational nitazene use in Moscow in 1998 resulting in 10 deaths and an isolated case of personal manufacture in Utah in 2003 [5]. Their emergence onto the illicit street market in recent years represents a concerning trend. The first nitazene to be widely identified in the recreational drug market was isotonitazene, in 2019, in Europe, Canada, and the United States. Following its international scheduling in 2021, a rapid proliferation of other nitazene analogues has emerged worldwide, with dozens of different nitazene analogues now reported in both toxicology investigations and seized-drug casework [6,7,8,9]. The potency of nitazenes is a major concern, as many analogues are substantially more potent than morphine and, in some cases, more potent than fentanyl. For instance, etonitazene has been described as 1000-fold more potent than morphine and up to 10–20 times more potent than fentanyl, while isotonitazene is reportedly 500 times more potent than morphine. This extreme potency means that even minimal quantities can cause intoxications and deadly overdoses, exacerbating the ongoing opioid crisis in regions like North America, Europe, and Australia [10,11,12].

Three main factors contribute to the rise of nitazenes [7,13]:

1. **Ease of synthesis**: Nitazene analogues are relatively straightforward to synthesise, do not require controlled precursors, and can be produced in small-scale clandestine laboratories worldwide.
2. **Economic appeal**: Their high potency means a few grams can yield thousands of doses, making them easier to conceal and transport, which is economically advantageous for traffickers.
3. **Regulatory evasion**: The rapid and consistent emergence of novel nitazene analogues with slight chemical modifications enables them to circumvent existing drug laws, which are often based on specific substances, creating a regulatory lag. This trend intensified after class-wide bans on fentanyl and its analogues in the late 2010s, prompting illicit chemists to seek alternative NSOs from the historical pharmacological literature.

**Figure 2:**
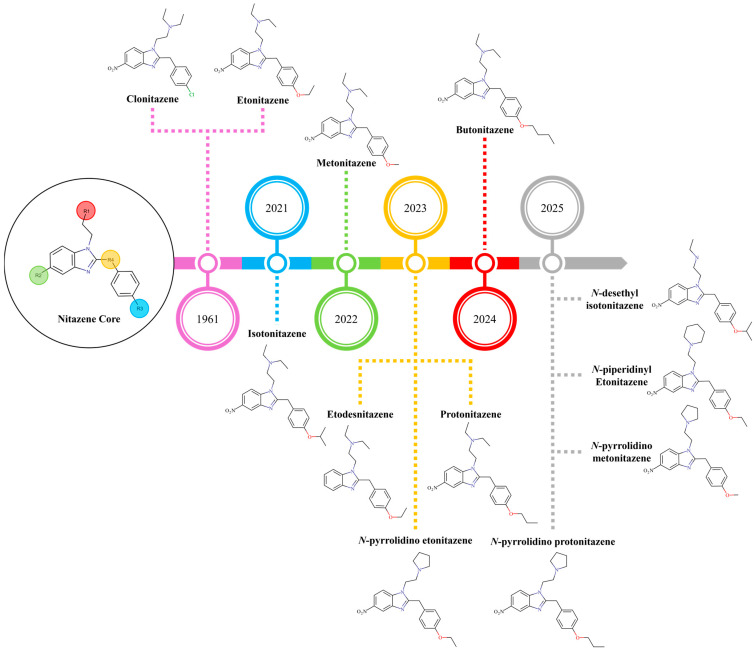
Nitazene core structure and 12 analogues under the UNODC International Drug Control Conventions [17]. Marvin was used for drawing the chemical structures: Marvin 17.21.0, Chemaxon (https://www.chemaxon.com).

Nitazenes are frequently found mixed with other drugs such as fentanyl, heroin, benzodiazepines, cocaine, and methamphetamine or mis-sold as counterfeit prescription medications like hydromorphone or oxycodone. This polysubstance use and misrepresentation result in unintentional exposure, particularly among opioid-naïve individuals, dramatically increasing the risk of overdose mortality [14,15,16]. The Centres for Disease Control and Prevention website even yielded zero results for nitazene in a 2022 search, highlighting a significant lack of awareness among clinicians, public health officials, and the general public [4]. The first two nitazenes, etonitazene and clonitazene, were placed under international control in 1961. More recently, around 30 additional nitazenes have been reported through the UNODC Early Warning Advisory System. To date, the United Nations Commission on Narcotic Drugs has scheduled 12 nitazenes, shown in Figure 2, under the International Drug Control Conventions [17].

## 2. The Situation in the World: A Growing Concern

**Figure 3:**
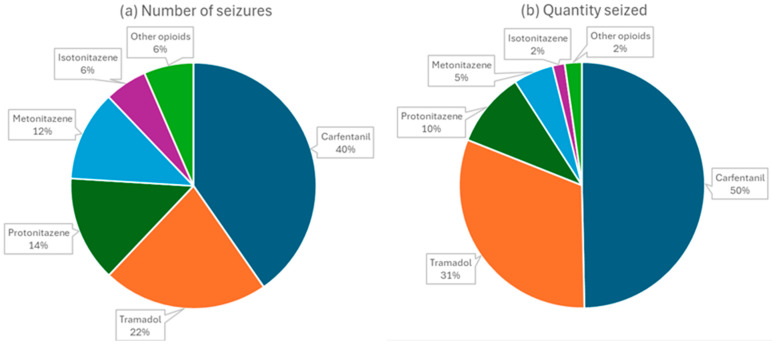
Top new opioids seized (**a**) by number of seizures (749 seizures) and (**b**) by quantity seized (16.6 kg seized) reported to the EU Early Warning System, 2022. Adapted from EUDA [26].

The global proliferation of nitazenes is currently a major public health concern, with their extreme potency posing a severe risk for overdose and mortality [18,19]. Nitazenes are increasingly identified in toxicology reports and recorded in cause-of-death fields on death certificates across multiple regions, including North America, Europe, Oceania, South America, and Southeast Asia [20,21]. In the United States, nitazenes have been implicated in overdose deaths in several states, with Tennessee identifying 52 nitazene-involved fatal drug overdoses from 2019 to 2021, showing a fourfold increase from 10 cases in 2020 to 42 in 2021. In 2021, metonitazene accounted for 85.7% of these deaths in Tennessee, despite being less potent than other nitazenes [22]. In Europe, 21 out of 27 EU Member States, as well as Norway, have reported the identification of nitazene opioids since 2019, with a rapid escalation in opioid-related deaths in some Baltic countries since 2022 [23]. The UK National Crime Agency reported 54 nitazene-related deaths within six months, a scenario likely to be an underestimation [19]. In Australia, 17 deaths have been attributed to nitazenes since 2021, alongside detections in emergency departments and drug seizures [24,25]. These facts are also confirmed by the European Union Drugs Agency (EUDA) in its latest European Drug Report [26], which states that in 2024, seven new synthetic opioids were formally reported to the EU Early Warning System, all of them nitazenes, the highest number reported in a single year. Since 2019, at least 21 EU Member States have reported the presence of a nitazene compound, and to date, 22 nitazenes have been detected in Europe. Furthermore, in 2023, the amount of nitazene powder seized in Europe tripled to 10 kg compared to 2022, alongside a significant increase in falsified medicines containing nitazene opioids. Within the group of synthetic opioids, the number of nitazenes reported to the EUDA, for the first time, has continued to rise, surpassing even fentanyl derivatives. Figure 3 shows the main opioids seized and reported to the EU Early Warning System, with nitazenes representing a considerable proportion.

A major concern is the unintentional consumption of nitazenes, as they are often mixed with or sold as other drugs such as heroin, fentanyl, benzodiazepines, methamphetamine, cocaine, or counterfeit medications (e.g., oxycodone, hydromorphone, Xanax, Dilaudid). Many users remain unaware they are taking nitazenes, markedly increasing the risk of overdose, particularly among opioid-naïve individuals. This polysubstance use, often involving combinations with fentanyl, methamphetamine, amphetamine, or flualprazolam, further complicates clinical management and increases the risk of synergistic respiratory depression [7,12,27].

The global opioid crisis, particularly in North America, has been primarily driven by fentanyl and other synthetic opioids. Nitazenes have the potential of becoming the drugs of choice in the future, resembling the shift that occurred with fentanyl [27]. This concern is exacerbated by factors such as the economic advantages of synthetic opioids, industrialised production, and regulatory crackdowns on fentanyl in China, which incentivise the development and distribution of even more potent substances [9]. This trend is consistent with the “Iron Law of Prohibition,” where efforts to suppress illicit drug supply lead to the emergence of more compact and potent substitutes. Additionally, the Taliban’s ban on opium cultivation in Afghanistan may create a void in the heroin market, potentially facilitating greater nitazene dissemination across Europe [23,28].

## 3. Pharmacological Profile and Toxicity of Nitazenes

Nitazenes are a class of NSOs that are chemically distinct from traditional opiates such as morphine and synthetic opioids like fentanyl and are characterised by a 2-benzylbenzimidazole core structure. This structural uniqueness contributes to their potent pharmacological effects. Nitazenes act as highly potent and selective μ-opioid receptor (MOR) agonists, the primary site of action for therapeutic and adverse opioid effects. They signal through both G protein-dependent and β-arrestin pathways, with many displaying superagonism at the MOR. Many nitazenes demonstrate MOR binding affinities comparable to, or exceeding, those of morphine and fentanyl [29,30,31,32,33].

The potency of nitazenes is exceptionally high, with some analogues being hundreds to thousands of times stronger than morphine, and many are comparable to or even exceed the potency of fentanyl. For instance, etonitazene has been reported to be 100–1000 times more potent than morphine and 10–20 times more potent than fentanyl [3,16], while isotonitazene is estimated at ~500 times the potency of morphine [12,34]. Metonitazene appears generally equipotent to fentanyl and ~100 times more potent than morphine, whereas protonitazene is ~2 times more potent than fentanyl and ~200 times more potent than morphine [3,12]. The metabolite *N*-desethyl isotonitazene has shown even greater potency than its parent drug, isotonitazene, and can induce longer-lasting respiratory depression compared to fentanyl. This combination of high potency and MOR superagonism can suggest the risk of prolonged hypoventilation and potential hypoxic injury [29,32,35].

Generally, nitazenes undergo rapid phase I biotransformation primarily via cytochrome P450 (CYP) enzymes, including CYP2D6, CYP2B6, and CYP2C8, producing metabolites through hydroxylation and dealkylation (e.g., *N*-desethylation, *O*-dealkylation). Phase II biotransformation occurs primarily by glucuronidation [36,37]. 4′-hydroxy-nitazene has been identified as a common metabolite of many nitazenes and may serve as a universal biomarker for their detection [13,21,33]. Since the metabolites can be even more potent than the parent compound, rapid metabolism does not necessarily represent a short duration of action.

The most critical and dangerous toxic effect of nitazenes is prolonged and severe respiratory depression, which is the primary cause of overdose deaths associated with these compounds [29]. In addition, nitazenes may carry cardiotoxic potential; computational analyses predict a high probability of hERG potassium channel inhibition, raising concerns about proarrhythmic effects [38,39,40]. Nitazenes exhibit a very narrow safe dose consumption range, meaning there is a small margin between the dose that provides desired effects and the dose that causes severe adverse effects such as respiratory depression or apnea. Like other opioids, nitazenes have a high potential for abuse and dependence through stimulation of the brain’s dopaminergic reward system [27,35].

Overdoses frequently involve polysubstance use, further complicating both clinical management and diagnosis [41], and in clinical settings, naloxone remains an effective antidote for nitazene intoxication, consistent with its efficacy against other potent opioids. However, due to their high potency, slow dissociation from the MOR and the prolonged effects of some active metabolites, higher or repeated doses of naloxone and, in some cases, continuous infusions may be required for complete reversal [11,42,43]. Reported overdose cases involving nitazenes have shown high naloxone dosing and relatively long hospital lengths of stay. Furthermore, the rapid metabolism of nitazenes and the involvement of polymorphic CYP enzymes may influence individual susceptibility to intoxication and addiction, while also complicating toxicological and forensic analysis [6,12,35,42].

## 4. Analytical Challenges in Forensic and Toxicological Investigations

Although nitazenes began appearing more consistently on the drug market in 2019, information on their pharmacology, toxicology, and associated harms remains limited. The rapid infiltration of nitazenes into the unregulated drug supply has also created significant analytical challenges for forensic and toxicological investigations [44,45].

One primary challenge is the inadequacy of traditional screening methods [27,46]. Immunoassays, routinely used for conventional opioids, are largely ineffective for most nitazenes due to their distinct chemical structures and insufficient cross-reactivity [47,48]. For example, studies confirm that commonly used fentanyl test strips fail to detect nitazenes. Although nitazene-specific test strips have recently become available, they present limitations, such as an inability to differentiate between nitazene analogues, potential issues with solubility in drug samples, and limited cross-reactivity with certain desnitazene analogues, which can give users a false sense of safety if not correctly interpreted [49]. Furthermore, older gas chromatography–mass spectrometry methods also frequently lack the necessary sensitivity for nitazenes, often missing them in casework [50,51,52]. From an analytical perspective, optimised sample preparation methods are essential for reliable nitazene detection. However, the chemical modifications within this class result in heterogeneous physicochemical properties; for example, some analogues are easily soluble in aqueous media while others are not, making method development even more challenging.

The extreme potency of nitazenes means they are typically present at very low concentrations in biological samples, often in the sub-ng/mL or sub-pmol/mL range, necessitating the development of highly sensitive analytical methods. Liquid chromatography–mass spectrometry-based techniques, such as liquid chromatography–triple-quadrupole mass spectrometry and liquid chromatography–time-of-flight mass spectrometry, have become the preferred approaches for screening and quantification in forensic and toxicological laboratories. These advanced systems can achieve the low limits of detection and quantification required. High-resolution mass spectrometry is particularly valuable for non-targeted screening and structural elucidation of new analogues [52,53,54,55,56]. Unfortunately, these instruments are very expensive to acquire and maintain, making them inaccessible to smaller laboratories and contributing to the underestimation of nitazene-related intoxications. This issue is even more pronounced in low-income countries, where adequate facilities and equipment are often lacking.

Another critical challenge is the constant emergence and structural modification of new nitazene analogues. This evolving context necessitates the continuous updating of analytical methods and spectral libraries to remain relevant. Additionally, newly emerging nitazene analogues, for which reference standards are not yet available, require additional experimental characterisation, including structural techniques such as nuclear magnetic resonance, which are not commonly accessible in routine forensic laboratories.

The structural similarities among many nitazene analogues, including numerous isomers, further complicate the identification process. Specialised chromatographic columns are crucial for achieving baseline separation of these isomers [55,57]. Techniques such as electron ionization–mass spectrometry, although often producing fragment-poor spectra for nitazenes, can differentiate analogues based on subtle differences in low-abundance fragment ions and typically require decision trees for interpretation [52]. Electron activated dissociation in ultra-high-performance liquid chromatography–quadrupole time-of-flight mass spectrometry is also being explored to elucidate fragmentation pathways for rapid structural identification [58]. Ion mobility spectrometry coupled with mass spectrometry shows promise for reference-free identification and isomer separation, potentially accelerating the detection of NSOs [59].

Although several methodologies for monitoring nitazenes have been reported in the literature [13], these methods must be continuously adjusted and validated in accordance with the equipment, protocols, regulations, and guidelines in each laboratory and country. The acquisition of the certified analytical standards presents an additional challenge, as they are expensive and sold in limited quantities, considering the amount of experimental work required to fully characterise each nitazene. Procuring these standards also involves time-consuming bureaucratic procedures. Given the high volume of samples routinely processed in forensic toxicology laboratories, developing new methods for nitazene detection is impractical, forcing outsourcing to specialised companies or academic institutions.

Polysubstance use is a common complicating factor, as nitazenes are rarely encountered in isolation. They are frequently mixed with other potent substances, including fentanyl, heroin, methamphetamine, benzodiazepines, and xylazine. This complex mixture complicates toxicological interpretation and clinical management, especially when users are unaware of nitazene exposure, thereby increasing the risk of severe overdose [41,60].

The identification and characterisation of nitazene metabolites are also crucial, as some metabolites (e.g., *N*-desethyl isotonitazene) can be pharmacologically active, in some cases more potent than their parent compounds, and may even emerge as standalone drugs [55,61]. Detecting these metabolites is very useful for forensic purposes, because they have a larger detection window and aid in identifying nitazene exposure when the parent compound is no longer present. However, a relatively common challenge in forensic toxicology laboratories is identifying the parent compound ingested when only common metabolites are detected in biological samples. For this reason, collaboration with first responder teams or police authorities is essential to verify whether any seized samples were found alongside the corpse so that they can be analysed and compared with the biological sample results. Forensic laboratories are also adapting to analyse nitazenes in alternative matrices such as hair [62] for retrospective exposure assessment, dried blood spots [54,63] for micro-sampling in clinical settings, and wastewater for early warning and epidemiological monitoring [50,64].

Another significant concern is the implication of nitazenes for animals, as studies of non-rodent species are extremely limited. Due to their high potency and lack of monitoring, nitazenes could potentially be involved in animal poisoning or accidental ingestions and remain undetected. In some Arabic countries, these compounds are used in camel racing as doping agents [65,66]. This emphasises the urgent need to develop analytical methodologies capable of detecting nitazenes in different matrices, either non-biological or biological, a task that remains very challenging and demanding.

Based on current evidence, nitazenes pose risks not only to humans but also to other animals and potentially to plants, demonstrating the need to include these compounds in a broader range of toxicological investigations.

## 5. Future Directions and Strategic Responses

The emergence of nitazenes, a class of potent synthetic opioids structurally distinct from fentanyl, represents a significant and escalating global public health challenge. Their increasing identification in the illicit drug supply worldwide, including in Europe, North America, Oceania, South America, and Southeast Asia, indicates the need for robust and proactive strategic responses. These novel substances present unique challenges in detection, clinical management, and public awareness, highlighting critical gaps in current opioid crisis responses [17,26,67]. Addressing the nitazene challenge requires a multifaceted approach that focuses on improved detection, enhanced clinical management, robust public health initiatives, and targeted research.

- Enhanced detection and surveillance:

Developing rapid and comprehensive testing methods is paramount. This includes creating new opioid test strips specifically capable of detecting nitazenes and improving laboratory techniques such as chromatography and high-resolution mass spectrometry to accurately identify and differentiate nitazene isomers. Monitoring common metabolites such as 4′-hydroxy-nitazene in urine offers a promising approach for routine and stable screening of various nitazene analogues, even when the parent compound is absent, thereby enhancing early identification [21]. Continuous development of analytical methodologies and vigilance toward newly emerging analogues in both European and non-European drug markets remain critical for effective surveillance.

Strengthening drug monitoring programs such as Australia’s Emerging Drugs Network and ensuring timely, collaborative information sharing across local and international jurisdictions (e.g., the UNODC Early Warning Advisory, the European Union Drugs Agency) are critical for issuing rapid public alerts about contaminated drug supplies and emerging nitazene variants. Fixed and event-based drug checking services, equipped with sensitive instruments capable of detecting low concentrations of nitazenes in various substances, including non-opioids, are essential for identifying unintentional contamination and informing harm reduction strategies [16,21].

Additionally, implementing rapid and simple nitazene-specific screening methods, for biological and/or non-biological samples, such as colorimetric assays, test strips, or sensor-based technologies, could be valuable in hospital emergency rooms, driving-under-the-influence police operations, and workplace healthcare. In hospitals, this would be particularly important for guiding timely treatment decisions, as current opioid screening methods may yield negative results even when a patient is experiencing opioid toxicity.

- Optimised clinical management:

Emergency physicians and first responders need comprehensive training on identifying nitazene intoxication, which can mimic other opioid overdoses, and to implement appropriate treatment protocols. Although naloxone is effective in reversing nitazene poisoning, specific guidance is needed regarding the minimum effective dose and the necessity of repeat or continuous administration due to prolonged drug and metabolite effects. Such recommendations should be informed by studies of monointoxications. Given the long duration of action of some nitazenes and their active metabolites, patients recovering from overdose may require prolonged hospitalisation and observation, potentially necessitating naloxone infusions. This contrasts with current guidelines for shorter observation periods for other opioids [12].

- Proactive public health and harm reduction:

Targeted public awareness campaigns are vital to inform the public, especially those who use stimulants or obtain pharmaceutical opioids outside medical channels, about the dangers of nitazene contamination. Expanding access to take-home naloxone kits in diverse settings, including entertainment venues, and promoting their use among all populations at risk, including opioid-naïve individuals, is crucial to reduce overdose fatalities and emphasise its life-saving potential [11,12,16,21].

Through the demystification of misconceptions about drug checking services and promoting their visibility, together with syringe residue analysis and wastewater monitoring programs, it becomes possible to better track circulating nitazenes among users and, simultaneously, implement harm reduction initiatives. These findings can also support drug outbreaks fingerprinting, helping to identify the origin of the samples. In collaboration with law enforcement authorities, such analyses can aid in disrupting trafficking routes and dismantling criminal networks. International cooperation and coordinated law enforcement efforts are crucial for combating the production and global distribution of nitazenes, which are frequently manufactured in countries like China and India and smuggled worldwide [26,68].

Educating the general public about nitazenes, including their composition, common excipients, and adulterants involved in their production, and the dangers associated with their consumption, can serve as an effective harm reduction strategy. For example, seminars targeting high school and university students, a key demographic for preventing initiation of drug use, may help reduce the number of new users. Similar educational initiatives have been successfully implemented in tobacco prevention programs, demonstrating promising results [69]. Although these are only strategies to prevent potential abuse, based essentially on chemical and toxicological perspectives, it is also important to conduct sociological and psychological studies to better understand consumption patterns and motivations.

- Focused research and policy development:

Further research is essential to characterise nitazene receptor kinetics, human pharmacokinetics, and the activity of their metabolites. Studies on monointoxications are critical to elucidate the isolated effects of these compounds. Exploration into novel opioid antagonists with prolonged efficacy could offer improved treatment options for persistent opioid toxicity. Additionally, implementing standardised reporting for overdose cases will enable better data collection and support future meta-analyses and evidence-based guideline development [12,21,67,70].

Continued efforts to place nitazenes under international control, such as their classification as Schedule I substances, are essential for limiting their global spread. Nowadays, with advances in forensic intelligence and artificial intelligence tools, it is possible to predict emerging nitazene analogues, as demonstrated in recent studies [30,39], and to understand their synthesis strategies and potential trafficking routes [71].

Finally, another measure adopted in some countries that could be implemented globally is the ban of known precursors chemicals. Rather than scheduling individual nitazenes under UNODC or other organisations laws, legislation could target the entire class of nitazene analogues, considering all possible chemical modifications of the core structure. Notwithstanding, it is important to note that while such measures may reduce the number of nitazenes in circulation, new families of psychoactive substances are likely to emerge rapidly to circumvent these regulations [1,2].

## 6. Conclusions

The emergence of nitazenes represents a rapidly escalating global public health threat distinct from previous opioid crises due to their extreme potency and structural diversity. Addressing this complex challenge demands a multifaceted and coordinated response. Priorities include the development of nitazene-specific rapid tests and advanced high-resolution mass spectrometry methodology to detect trace concentrations and differentiate isomers, as well as monitoring common metabolites such as 4′-hydroxy-nitazene for early identification. In clinical practice, comprehensive training for emergency personnel and updated guidance on naloxone dosing are essential, as higher, repeated, or continuous administration may be required. Public health strategies should incorporate targeted awareness campaigns and wider distribution of take-home naloxone kits across diverse at-risk populations. At the same time, focused research on pharmacology, metabolites, and novel antagonists, combined with class-wide legislative controls and international collaboration between law enforcement, universities, and drug-checking associations to disrupt trafficking routes, will be vital to reduce harms. Only through these concerted measures can the growing impact of nitazenes be effectively mitigated.

## Data Availability

Data sharing is not applicable.

## References

[1] United Nations Office on Drugs and Crime (2025). World Drug Report 2025. https://www.unodc.org/unodc/data-and-analysis/world-drug-report-2025.html.

[2] European Union Drugs Agency (2025). European Drug Report 2025 Trends and Developments. https://www.euda.europa.eu/publications/european-drug-report/2025_en.

[3] Lassi N., Jiang S. (2025). The Future of Deadly Synthetic Opioids: Nitazenes and Their International Control. Glob. Policy.

[4] Pergolizzi J., Raffa R., LeQuang J.A.K., Breve F., Varrassi G. (2023). Old Drugs and New Challenges: A Narrative Review of Nitazenes. Cureus.

[5] Advisory Council on the Misuse of Drugs (2025). ACMD Advice on 2-Benzyl Benzimidazole and Piperidine Benzimidazolone Opioids (Accessible Version). https://www.gov.uk/government/publications/acmd-advice-on-2-benzyl-benzimidazole-and-piperidine-benzimidazolone-opioids/acmd-advice-on-2-benzyl-benzimidazole-and-piperidine-benzimidazolone-opioids-accessible-version.

[6] Isoardi K.Z., Alfred S., Weber C., Harris K., Soderstrom J., Syrjanen R., Thompson A., Schumann J., Stockham P., Sakrajda P. (2025). Clinical Toxicity of Nitazene Detections in Two Australian Emergency Department Toxicosurveillance Systems. Drug Alcohol. Rev..

[7] Dugues P., Rabai A., Chenorhokian S., Pfau G., Cherki S., Bellouard M., Alvarez J.C., Larabi I.A. (2025). Emergence of Counterfeit Oxycodone Tablets Containing Nitazenes in France: First National Alert and Analytical Characterization. Toxicol. Anal. Clin..

[8] Gonçalves de Araújo K.R., Fabris A.L., Junior L.F.N., Soares A.L., Costa J.L., Yonamine M. (2024). Synthetic Illicit Opioids in Brazil: Nitazenes Arrival. Forensic Sci. Int. Rep..

[9] Meyer M., Westenberg J.N., Jang K.L., Choi F., Schreiter S., Mathew N., King C., Lang U.E., Vogel M., Krausz R.M. (2023). Shifting Drug Markets in North America—A Global Crisis in the Making?. Int. J. Ment. Health Syst..

[10] Glatfelter G.C., Vandeputte M.M., Chen L., Walther D., Tsai M.H.M., Shi L., Stove C.P., Baumann M.H. (2023). Alkoxy Chain Length Governs the Potency of 2-Benzylbenzimidazole ‘Nitazene’ Opioids Associated with Human Overdose. Psychopharmacology.

[11] Berger J.C., Severe A.D., Jalloh M.S., Manini A.F. (2025). Naloxone Dosing and Hospitalization for Nitazene Overdose: A Scoping Review. J. Med. Toxicol..

[12] Stangeland M., Dale O., Skulberg A.K. (2025). Nitazenes: Review of Comparative Pharmacology and Antagonist Action. Clin. Toxicol..

[13] Vandeputte M.M., Stove C.P. (2025). Navigating Nitazenes: A Pharmacological and Toxicological Overview of New Synthetic Opioids with a 2-Benzylbenzimidazole Core. Neuropharmacology.

[14] Monti M.C., De Vrieze L.M., Vandeputte M.M., Persson M., Gréen H., Stove C.P., Schlotterbeck G. (2024). Detection of N-Desethyl Etonitazene in a Drug Checking Sample: Chemical Analysis and Pharmacological Characterization of a Recent Member of the 2-Benzylbenzimidazole “Nitazene” Class. J. Pharm. Biomed. Anal..

[15] Keller E.L., Peake B., Simpson B.S., Longo M., Trobbiani S., White J.M., Gerber C. (2025). Searching for a Needle in a Haystack: Chemical Analysis Reveals Nitazenes Found in Drug Paraphernalia Residues. Drug Alcohol. Rev..

[16] Mammoliti E., Nielsen S., Roxburgh A. (2025). A Scoping Review of the Emergence of Novel Synthetic Opioids in Australian Drug Markets: What Does This Mean for Harm Reduction Responses?. Drug Alcohol. Rev..

[17] United Nations Office on Drugs and Crime (2025). February 2025-UNODC EWA: Increasing Availability of Nitazenes Calls for Global Response. https://www.unodc.org/LSS/Announcement/Details/b47cf39e-f557-4001-98a8-536af5673e9e.

[18] Schumann J.L., Dwyer J., Brown J.A., Jauncey M., Roxburgh A. (2025). Identification of Nitazene-Related Deaths in Australia: How Do We Make It Accurate and Timely?. Drug Alcohol. Rev..

[19] Holland A., Copeland C.S., Shorter G.W., Connolly D.J., Wiseman A., Mooney J., Fenton K., Harris M. (2024). Nitazenes—Heralding a Second Wave for the UK Drug-Related Death Crisis?. Lancet Public Health.

[20] Lo Faro A.F., Berardinelli D., Cassano T., Dendramis G., Montanari E., Montana A., Berretta P., Zaami S., Busardò F.P., Huestis M.A. (2023). New Psychoactive Substances Intoxications and Fatalities during the COVID-19 Epidemic. Biology.

[21] Verbeek J., Brinkman D.J. (2025). A Comprehensive Narrative Review of Protonitazene: Pharmacological Characteristics, Detection Techniques, and Toxicology. Basic. Clin. Pharmacol. Toxicol..

[22] Roberts A., Korona-Bailey J., Mukhopadhyay S. (2022). Nitazene-Related Deaths—Tennessee, 2019–2021. Morb. Mortal. Wkly. Rep..

[23] Griffiths P.N., Seyler T., De Morais J.M., Mounteney J.E., Sedefov R.S. (2024). Opioid Problems Are Changing in Europe with Worrying Signals That Synthetic Opioids May Play a More Significant Role in the Future. Addiction.

[24] Darke S., Duflou J., Farrell M., Lappin J., Peacock A. (2024). Emergence of Deaths Due to Nitazene Toxicity in Australia. Drug Alcohol Rev..

[25] Oelrichs R. (2025). A National Perspective. Med. J. Aust..

[26] European Union Drugs Agency (2025). EU Drug Market: New Psychoactive Substances-Distribution and Supply in Europe: New Opioids. https://www.euda.europa.eu/publications/eu-drug-markets/new-psychoactive-substances/distribution-and-supply/new-opioids_en.

[27] Zawilska J.B., Adamowicz P., Kurpeta M., Wojcieszak J. (2023). Non-Fentanyl New Synthetic Opioids—An Update. Forensic Sci. Int..

[28] Giommoni L. (2024). How to Improve the Surveillance of the Taliban Ban’s Impact on European Drug Markets. Int. J. Drug Policy.

[29] Malcolm N.J., Palkovic B., Sprague D.J., Calkins M.M., Lanham J.K., Halberstadt A.L., Stucke A.G., McCorvy J.D. (2023). Mu-Opioid Receptor Selective Superagonists Produce Prolonged Respiratory Depression. iScience.

[30] Vandeputte M.M., Glatfelter G.C., Walther D., Layle N.K., St. Germaine D.M., Ujváry I., Iula D.M., Baumann M.H., Stove C.P. (2024). Characterization of Novel Nitazene Recreational Drugs: Insights into Their Risk Potential from in Vitro µ-Opioid Receptor Assays and in Vivo Behavioral Studies in Mice. Pharmacol. Res..

[31] Tsai M.H.M., Chen L., Baumann M.H., Canals M., Javitch J.A., Lane J.R., Shi L. (2024). In Vitro Functional Profiling of Fentanyl and Nitazene Analogs at the μ-Opioid Receptor Reveals High Efficacy for Gi Protein Signaling. ACS Chem. Neurosci..

[32] De Vrieze L.M., Walton S.E., Pottie E., Papsun D., Logan B.K., Krotulski A.J., Stove C.P., Vandeputte M.M. (2024). In Vitro Structure–Activity Relationships and Forensic Case Series of Emerging 2-Benzylbenzimidazole ‘Nitazene’ Opioids. Arch. Toxicol..

[33] Magny R., Schiestel T., M’Rad A., Lefrère B., Raphalen J.H., Ledochowski S., Labat L., Mégarbane B., Houzé P. (2025). Comparison of the Metabolic Profiles Associated with Protonitazene and Protonitazepyne in Two Severe Poisonings. Metabolites.

[34] Tomiyama K.I., Funada M. (2025). The Synthetic Opioid Isotonitazene Induces Locomotor Activity and Reward Effects through Modulation of the Central Dopaminergic System in Mice. Toxicol. Appl. Pharmacol..

[35] Baldo B.A. (2025). Opioid-Induced Respiratory Depression: Clinical Aspects and Pathophysiology of the Respiratory Network Effects. Am. J. Physiol. Lung Cell Mol. Physiol..

[36] Jadhav G.R., Fasinu P.S. (2024). Metabolic Characterization of the New Benzimidazole Synthetic Opioids—Nitazenes. Front. Pharmacol..

[37] Ameline A., Gheddar L., Pichini S., Stove C., Aknouche F., Maruejouls C., Raul J.S., Kintz P. (2024). In Vitro Characterization of Protonitazene Metabolites, Using Human Liver Microsomes, and First Application to Two Urines Collected from Death Cases. Clin. Chim. Acta.

[38] Hancox J.C., Wang Y., Copeland C.S., Zhang H., Harmer S.C., Henderson G. (2024). Nitazene Opioids and the Heart: Identification of a Cardiac Ion Channel Target for Illicit Nitazene Opioids. J. Mol. Cell. Cardiol. Plus.

[39] Clayton J., Shi L., Robertson M.J., Skiniotis G., Michaelides M., Stavitskaya L., Shen J. (2025). A Putative Binding Model of Nitazene Derivatives at the μ-Opioid Receptor. Neuropharmacology.

[40] Hataoka K., Hojo M., Nomura S., Nakagawa Y., Kawai A., Nakamura M., Ikushima K., Alexander D.B., Suzuki J., Suzuki T. (2025). Evaluation of Rewarding Effects of Nitazene Analogs: Results from Conditioned Place Preference Tests and in Vivo Microdialysis Experiments in Mice. J. Toxicol. Sci..

[41] Pucci M., Singh Jutley G., Looms J., Ford L. (2024). N-Desethyl Isotonitazene Detected in Polydrug Users Admitted to Hospital in Birmingham, United Kingdom. Clin. Toxicol..

[42] Amaducci A., Aldy K., Campleman S.L., Li S., Meyn A., Abston S., Culbreth R.E., Krotulski A., Logan B., Wax P. (2023). Naloxone Use in Novel Potent Opioid and Fentanyl Overdoses in Emergency Department Patients. JAMA Netw. Open.

[43] Alhosan N., Cavallo D., Santiago M., Kelly E., Henderson G. (2025). Slow Dissociation Kinetics of Fentanyls and Nitazenes Correlates with Reduced Sensitivity to Naloxone Reversal at the μ-Opioid Receptor. Br. J. Pharmacol..

[44] Bade R., Nadarajan D., Driver E.M., Halden R.U., Gerber C., Krotulski A., Hall W., Mueller J.F. (2024). Wastewater-Based Monitoring of the Nitazene Analogues: First Detection of Protonitazene in Wastewater. Sci. Total Environ..

[45] Curtis B., Lawes D.J., Caldicott D., McLeod M.D. (2025). Identification of the Novel Synthetic Opioid N-Pyrrolidino Isotonitazene at an Australian Drug Checking Service. Drug Test. Anal..

[46] Palmquist K.B., Truver M.T., Shoff E.N., Krotulski A.J., Swortwood M.J. (2023). Review of Analytical Methods for Screening and Quantification of Fentanyl Analogs and Novel Synthetic Opioids in Biological Specimens. J. Forensic Sci..

[47] Sisco E., Appley M.G., Pyfrom E.M., Banta-Green C.J., Shover C.L., Molina C.A., Biamont B., Robinson E.L. (2024). Beyond Fentanyl Test Strips: Investigating Other Urine Drug Test Strips for Drug Checking Applications. Forensic Chem..

[48] Pacana A.L., Skillman B.N. (2025). Evaluation of Enzyme-Linked Immunosorbent Assay Screening Kits for the Detection of Nitazene Analogs. J. Forensic Sci..

[49] De Vrieze L.M., Stove C.P., Vandeputte M.M. (2024). Nitazene Test Strips: A Laboratory Evaluation. Harm Reduct. J..

[50] Keller E.L., Peake B., Simpson B.S., White J.M., Gerber C. (2025). Comprehensive Method to Detect Nitazene Analogues and Xylazine in Wastewater. Environ. Sci. Pollut. Res..

[51] Phelps C., Hardwick E.K., Couch A.N., Davidson J.T. (2025). Development and Validation of a Combined Selected Ion Monitoring-Scan GC-EI-MS Method for Nitazene Analogs. J. Forensic Sci..

[52] Hardwick E.K., Tyler Davidson J. (2024). Structural Characterization of Nitazene Analogs Using Electron Ionization-Mass Spectrometry (EI-MS). Forensic Chem..

[53] Hardwick E.K., Davidson J.T. (2025). Structural Characterization of Nitazene Analogs Using Electrospray Ionization–Tandem Mass Spectrometry (ESI–MS/MS). Drug Test. Anal..

[54] Ververi C., Galletto M., Massano M., Alladio E., Vincenti M., Salomone A. (2024). Method Development for the Quantification of Nine Nitazene Analogs and Brorphine in Dried Blood Spots Utilizing Liquid Chromatography—Tandem Mass Spectrometry. J. Pharm. Biomed. Anal..

[55] Wachełko O., Tusiewicz K., Szpot P., Zawadzki M. (2025). The UHPLC-MS/MS Method for the Determination of 26 Synthetic Benzimidazole Opioids (Nitazene Analogs) with Isomers Separation. J. Pharm. Biomed. Anal..

[56] Schüller M., Lucic I., Øiestad Å.M.L., Pedersen-Bjergaard S., Øiestad E.L. (2023). High-Throughput Quantification of Emerging “Nitazene” Benzimidazole Opioid Analogs by Microextraction and UHPLC-MS-MS. J. Anal. Toxicol..

[57] Gao G., Yang S., Wang X., Xiang P., Ma L., Yan F., Shi Y. (2025). UHPLC-MS/MS-Based Analysis of 17 Nitazenes in Human Hair for Practical Forensic Casework with Simultaneous Separation of 6 Groups of Isomers. J. Pharm. Biomed. Anal..

[58] Liu C.M., Huang B.Y., Hua Z.D., Jia W., Li Z.-Y. (2025). Characterization of Mass Spectrometry Fragmentation Patterns Under Electron-Activated Dissociation (EAD) for Rapid Structure Identification of Nitazene Analogs. Rapid Commun. Mass. Spectrom..

[59] Hollerbach A.L., Lin V.S., Ibrahim Y.M., Ewing R.G., Metz T.O., Rodda K.E. (2024). Elucidating the Gas-Phase Behavior of Nitazene Analog Protomers Using Structures for Lossless Ion Manipulations Ion Mobility-Orbitrap Mass Spectrometry. J. Am. Soc. Mass. Spectrom..

[60] Killoran S., McNamara S., Kavanagh P., O’Brien J., Lakes R. (2025). Identification of N-Pyrrolidino Protonitazene in Powders Sold as Heroin and Associated with Overdose Clusters in Dublin and Cork, Ireland. Drug Test. Anal..

[61] Kriikku P., Pelander A., Jylhä A., Ojanperä I. (2025). Post-Mortem Identification and Toxicological Findings of Fluetonitazepyne and Isotonitazepyne. Drug Test. Anal..

[62] Kintz P., Ameline A., Gheddar L., Pichini S., Mazoyer C., Teston K., Aknouche F., Maruejouls C. (2024). Testing for Protonitazene in Human Hair Using LC–MS-MS. J. Anal. Toxicol..

[63] Pardi J., Ford S., Cooper G. (2023). Validation of an Analytical Method for Quantitation of Metonitazene and Isotonitazene in Plasma, Blood, Urine, Liver and Brain and Application to Authentic Postmortem Casework in New York City. J. Anal. Toxicol..

[64] Bade R., Nadarajan D., Hall W., Brown J.A., Schumann J. (2025). Early Identification of the Use of Potent Benzylbenzimidazoles (Nitazenes) through Wastewater Analysis: Two Years of Data from 22 Countries. Addiction.

[65] Nalakath J., Thacholil R.P., Kadry A.P.S., Praseen O.K. (2025). LCMS Detection and Characterization of In Vitro Metabolites of Isotonitazene, a Targeted Strategy for Novel Psychoactive Substance Control in Camel Racing. Rapid Commun. Mass. Spectrom..

[66] Nalakath J., Palathinkal A.B., Naduvilakkandy R., Vazhat R.A., Komathu P.O. (2025). In Vitro Metabolism of Metonitazene in Camels: High-Resolution Mass Spectrometric Characterization for Doping Control. Rapid Commun. Mass. Spectrom..

[67] United Nations Office on Drugs and Crime (2024). The Challenge of New Psychoactive Substances—A Technical Update. https://www.unodc.org/unodc/en/scientists/the-challenge-of-new-psychoactive-substances.html.

[68] Bhuiyan I., Tobias S., Ti L. (2023). Responding to Changes in the Unregulated Drug Supply: The Need for a Dynamic Approach to Drug Checking Technologies. Am. J. Drug Alcohol. Abus..

[69] Jamal A., Park-Lee E., Birdsey J., West A., Cornelius M., Cooper M.R., Cowan H., Wang J., Sawdey M.D., Cullen K.A. (2024). Morbidity and Mortality Weekly Report Tobacco Product Use Among Middle and High School Students-National Youth Tobacco Survey, United States, 2024. Weekly.

[70] Kozell L.B., Eshleman A.J., Wolfrum K.M., Swanson T.L., Schutzer K.A., Schutzer W.E., Abbas A.I. (2025). Pharmacology of Newly Identified Nitazene Variants Reveals Structural Determinants of Affinity, Potency, Selectivity for Mu Opioid Receptors. Neuropharmacology.

[71] Gray A., Douglas S., Tiller M., Bleakley M. (2024). Using Forensic Intelligence as a Model for Determining Future Toxicology Methods: TBI Forensic Toxicology and Forensic Drug Chemistry Nitazene Identification. J. Anal. Toxicol..

